# The systemic pathology of cerebral malaria in African children

**DOI:** 10.3389/fcimb.2014.00104

**Published:** 2014-08-21

**Authors:** Danny A. Milner, Richard O. Whitten, Steve Kamiza, Richard Carr, George Liomba, Charles Dzamalala, Karl B. Seydel, Malcolm E. Molyneux, Terrie E. Taylor

**Affiliations:** ^1^Department of Pathology, Brigham and Women's HospitalBoston, MA, USA; ^2^Department of Immunology and Infectious Disease, Harvard School of Public HealthBoston, MA, USA; ^3^The Blantyre Malaria Project, College of Medicine, University of MalawiBlantyre, Malawi; ^4^CellNetix Pathology and LaboratoriesOlympia, Washington, USA; ^5^Department of Histopathology, College of Medicine, University of MalawiBlantyre, Malawi; ^6^Department of Histopathology, South Warwickshire General HospitalsWarwick, UK; ^7^Department of Osteopathic Medical Specialties, College of Osteopathic Medicine, Michigan State UniversityEast Lansing, MI, USA; ^8^Malawi-Liverpool-Wellcome Trust Clinical Research Programme, College of MedicineBlantyre, Malawi; ^9^Liverpool School of Tropical Medicine, University of LiverpoolLiverpool, UK

**Keywords:** autopsy, cerebral malaria, pediatrics, Africa, Malawi, pathology

## Abstract

Pediatric cerebral malaria carries a high mortality rate in sub-Saharan Africa. We present our systematic analysis of the descriptive and quantitative histopathology of all organs sampled from a series of 103 autopsies performed between 1996 and 2010 in Blantyre, Malawi on pediatric cerebral malaria patients and control patients (without coma, or without malaria infection) who were clinically well characterized prior to death. We found brain swelling in all cerebral malaria patients and the majority of controls. The histopathology in patients with sequestration of parasites in the brain demonstrated two patterns: (a) the “classic” appearance (i.e., ring hemorrhages, dense sequestration, and extra-erythrocytic pigment) which was associated with evidence of systemic activation of coagulation and (b) the “sequestration only” appearance associated with shorter duration of illness and higher total burden of parasites in all organs including the spleen. Sequestration of parasites was most intense in the gastrointestinal tract in all parasitemic patients (those with cerebral malarial and those without).

## Introduction

Malaria is an ancient disease affecting 200 million people annually; most of the ~655,000 deaths each year occur in children under 5 years of age in sub-Saharan Africa (White, 2007; WHO Global Malaria Program, 2008, 2011). A common form of severe malaria is cerebral malaria, clinically defined as coma (Blantyre Coma Score ≤ 2) persisting despite correction of hypoglycemia or seizures, with parasitemia and no bedside evidence of an alternative cause of coma such as meningitis or post-ictal state clinical cerebral malaria (CCM). This standard clinical definition misclassifies 25–30% of patients, and can be improved by the addition of retinal examination to identify features specific for histologically defined cerebral malaria (HCM) (Karney and Tong, 1972; Lewallen et al., 1993, 1999, 2000; Beare et al., 2004, 2006; Taylor et al., 2004). The pathogenesis of cerebral malaria remains controversial, and the precise pathway from illness to death difficult to discern; evidence exists to suggest a variety of plausible pathways and a number of individual factors which could impinge upon those pathways (Toro and Roman, 1978; Looareesuwa et al., 1983; Gopinathan et al., 1986; Clark et al., 1987, 2003a,b; Oo et al., 1987; Aikawa, 1988; Pongponratn et al., 1991; Aikawa et al., 1992; Nagatake et al., 1992; Molyneux et al., 1993; Patnaik et al., 1994; Turner et al., 1994; Nakazawa et al., 1995; Lucas et al., 1997; Richards, 1997; Newton et al., 1998; Lou et al., 2001; Piguet et al., 2002; Clark and Cowden, 2003; Liechti et al., 2003; Lopansri et al., 2003; Pino et al., 2003, 2005; Dzeing-Ella et al., 2005; Maguire et al., 2005; Viebig et al., 2005; Wassmer et al., 2005; Kaestli et al., 2006; Montgomery et al., 2006, 2007; Seydel et al., 2006; Dondorp et al., 2008; Milner et al., 2008). Similarly, understanding the causes of mortality, including the etiology and significance of increased brain volume (e.g., cerebral edema vs. other causes), has not been straightforward (Spitz, 1946; MacPherson et al., 1985; Taylor et al., 2004; Milner et al., 2012, 2013a). The range of pathology as well as variability in mortality rates leads us to conclude that HCM (like CCM) is not a homogeneous syndrome but rather a heterogeneous collection along a spectrum of severe malaria with multiple points of variation. The last systematic autopsy study of severe malaria was done by Spitz (1946). She analyzed cases of adult American soldiers (all adults) returning from World War II, a group of travelers with no acquired immunity to malaria (Spitz, 1946). In 1985, MacPherson et al described cerebral ultrastructural findings in 7 Thai adults, in some of whom up to four other tissues were also examined (MacPherson et al., 1985). None of these studies was prospective. In addition, since 1954, not a single study has addressed the pathology outside of the brain in a comprehensive or prospective manner in children. In 1996, we initiated a prospective study of the clinicopathological correlates of fatal pediatric malaria, including cases and controls, in Blantyre Malawi. Nearly 2291 children were enrolled, of whom 404 died (335 for whom permission to conduct an autopsy was sought and 287 with clinical retinal examinations). We performed 103 autopsies, and here we summarize the pathology in all organs. We tested the following related hypotheses regarding the pathology of fatal cerebral malaria:

- Retinopathy positive CCM is a condition associated with a high burden of sequestration throughout the organs of the body, including the brain.
- Retinopathy negative CCM (i.e., comatose patients with parasitemia who do not have evidence of malarial retinopathy) have little to no burden of sequestered parasites (i.e., do not have HCM).

## Materials and methods

### Patients

**Figure 1:**
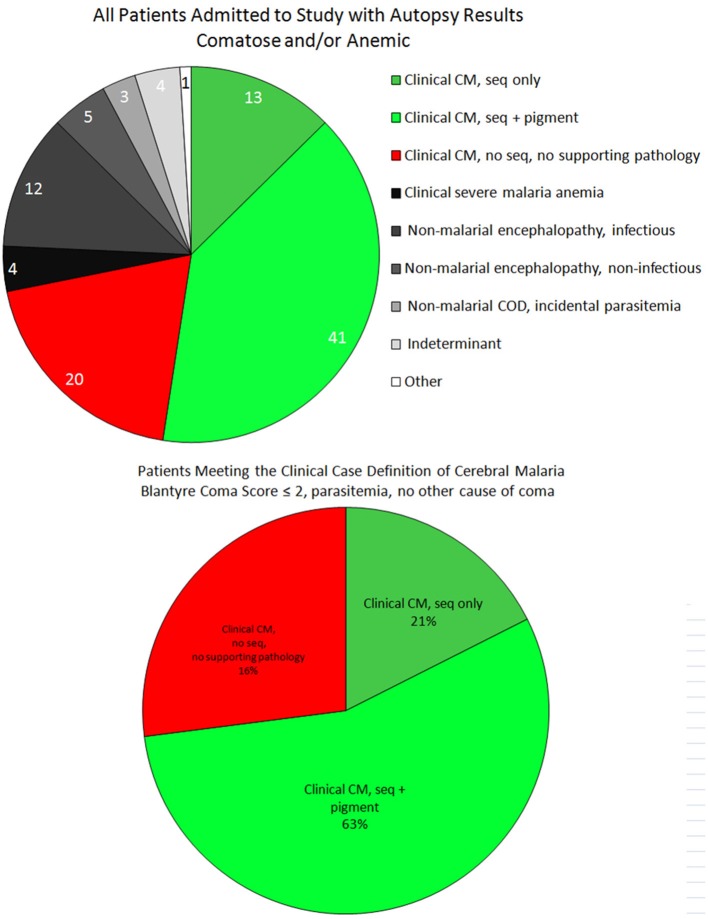
**The case distribution of all autopsies between 1996 and 2010 is presented for all final diagnoses (top)**. These included patients who were admitted to the research ward who were either comatose or anemic (with or without parasitemia). Among patients who satisfied the clinical case definition of CM (bottom), the majority were “classic” CM (CM2, ring hemorrhages and excessive pigment), followed by the “sequestration only” pattern (CM1, early disease). The remainder were the misclassified cases (CM3) in which a non-malaria cause of death was identified at autopsy.

The institutional review boards of Michigan State University, the University of Malawi College of Medicine, and Brigham and Women's Hospital reviewed all or parts of this study. Autopsies were requested by a Malawian clinician or nurse and, if parents consented, performed as quickly after death as possible in the morgue of the Queen Elizabeth Central Hospital in Blantyre, Malawi. Intervals between death and autopsy were noted. Patient recruitment, clinical management, autopsy protocols, and laboratory investigations have been previously described (Taylor et al., 2004). The definition of CCM in this study included a Blantyre coma score of ≤2, peripheral *P. falciparum* parasitemia, and no other obvious cause of coma (i.e., hypoglycemia, post-ictal state, or meningitis). Control patients consisted of those with *P. falciparum* infections who were not in coma [i.e., severe malarial anemia (SMA)], and those who were in comas not attributable to malaria (children in a coma without peripheral parasitemia, parasitemic individuals in whom non-malarial causes of coma were identified in life), indeterminant cases (children who died too quickly to establish a clinical diagnosis), and non-malarial deaths in children without coma or parasitemia (Figure 1). Time from illness to death were tested using non-parametric tests.

### Histological classification

Cases in whom the diagnosis of CCM was established according to traditional clinical criteria during life were classified, according to cerebral histopathological appearances and general autopsy findings, into the following HCM categories: CM1—CCM with sequestration of parasitized red blood cells (PRBCs) in the brain, no additional cerebral histopathological changes, and no other cause of death; CM2—CCM with sequestration of PRBCs in the brain and the presence of cerebral microthrombi, ring hemorrhages and extra-erythrocytic malaria pigment, and no other cause of death; CM3—fulfilling the traditional definition of CCM in life, but with no sequestration of PRBCs in the brain and another cause of death identified (Figure 1) (Taylor et al., 2004). Control cases were classified by the pathology responsible for death.

### Autopsy and histological assessment

At autopsy, all organs were weighed and photographed. The brain and all organs were dissected fresh. All histological tissues used in the study were fixed in fresh 10% neutral buffered formalin, processed, embedded in paraffin, sectioned at 3–4 microns and stained with hematoxylin and eosin per routine protocols. All organs were reviewed and described independently by at least two pathologists. For each organ for all cases, the pathologists were asked to score the vasculature for the presence or absence of sequestration based on the qualitative presence of parasites within capillaries and/or sinusoids.

## Results

Of the 103 patients who underwent autopsy, the final anatomical diagnoses were distributed as follows: CM1 = 13, CM2 = 41, CM3 = 20, SMA = 4, non-malarial coma = 17, incidental parasitemia = 3, indeterminant = 4, and non-malarial death = 1. For the first three groups (i.e., those patients meeting the criteria for CCM), the median time from admission to death was almost identical, while the time from onset of clinical symptoms to death was longer for CM2 patients compared to both CM1 and CM3 (Table 1).

**Table 1:**
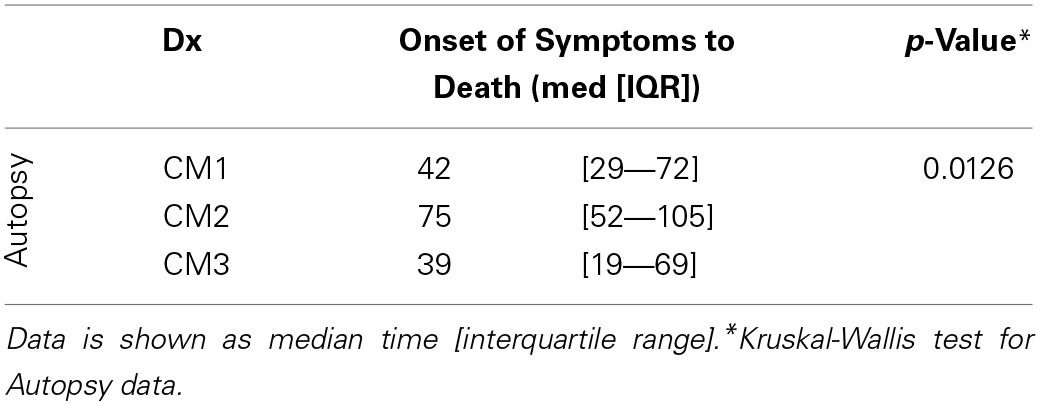
**The time (hours) from onset of symptoms (based on history from parents/guardians) to death for all fatal cases in the study for which there was autopsy (*n* = 103) data available demonstrate that CM1 cases appear to be a shorter duration of illness than CM2 patients consistent with our observations of the histopathology**.

Data is shown as median time [interquartile range].^*^Kruskal-Wallis test for Autopsy data.

In review, 2 of the 4 indeterminant cases could be classified as HCM but insufficient clinical data were gathered to make a pre-mortem diagnosis. The single non-malarial, non-comatose death was an aparasitemic patient with pneumonia.

### Central nervous system (CNS)

**Figure 2:**
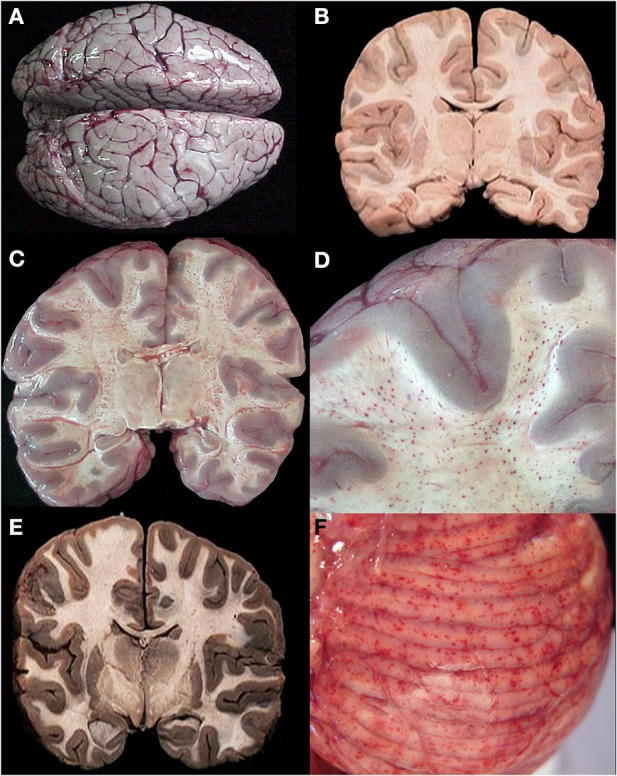
**A summary of representative images from the gross examination of the brain in the autopsy series is shown for CM cases**. The vast majority of cases, regardless of diagnosis, showed brain swelling with flattened gyri and narrowed sulci **(A)**. In this example, the brain has the classic “slate gray” to “purple” appearance of CM which is possibly due to malaria pigment within vessels. In all control cases and in a subset of the CM1 cases, the coronal brain slice appeared without discoloration **(B)**. In the classic CM2 appearance, petechial hemorrhages are seen diffusely in the white matter throughout the brain **(C)**. A higher magnification demonstrates the abrupt transition from white to gray matter and the lack of hemorrhages in the gray **(D)**. In a subset of the CM1 cases, the coronal brain slice appeared very discolored (an unexplained phenmenon) and swollen but without petechial hemorrhages in the cerebral cortex **(E)**. The cerebellum had petechial hemorrhages in both the gray and white matter and thus, visible on the surface grossly **(F)**. **(B,E)** Were previously published in part in the Journal of Infectious Diseases (2005) and appear here with express permission (see Milner et al., 2005; White and Silamut, 2005).

We sampled 18 locations in the CNS, 12 of which had consistent histological evidence of sequestration (italics): Cerebral cortex (*frontal, parietal, temporal, and occipital lobes), hippocampus*, *caudate nucleus, thalamus, midbrain, pons, medulla,* spinal cord, *cerebellar folia, cerebellar dentate nucleus*, choroid plexus in the lateral ventricles, optic nerve, pituitary, and sagittal and transverse sinuses. Gross examination of the brains of HCM patients (>21% of capillaries with sequestration) showed swelling and variable color from classic slate gray to dark purple (Figure 2A). Sinus thrombosis was not seen. Control cases and a few examples of CM1 had a relatively normal color to the brain while most CM2 cases were discolored (Figures 2B–E).

**Figure 3:**
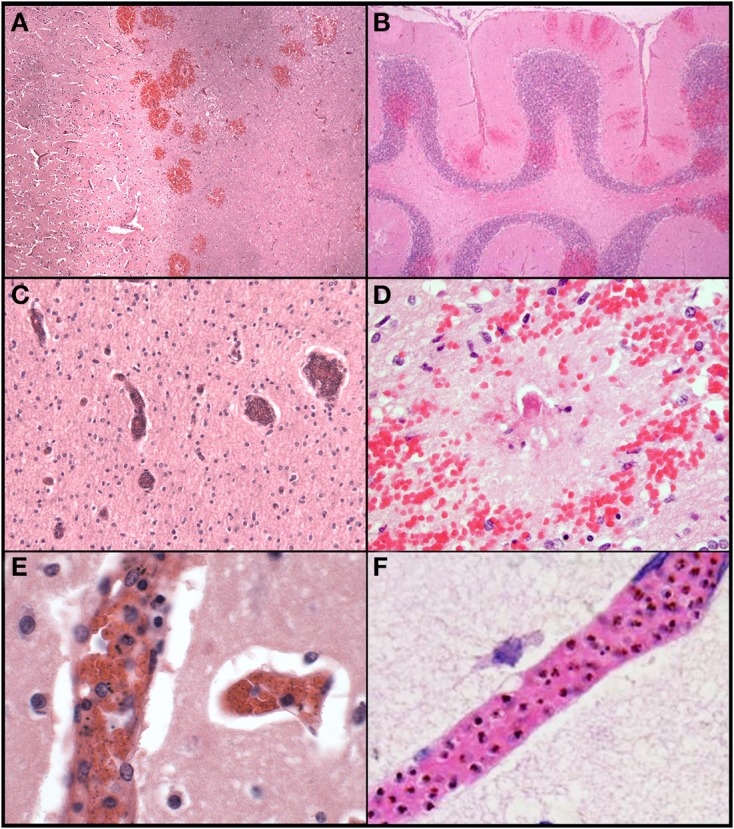
**A summary of representative images from the histological examination of the brain in the autopsy series is shown for CM cases**. The abrupt transition from gray to white matter **(A)** and the presence of ring hemorrhages are demonstrated in this classic case of CM (CM2, 100X, H&E). The cerebellum **(B)** with ring hemorrhages in all levels including white and gray matter are shown (100x, H&E). Visibly congested blood vessels **(C)** even at low power may be the result of dense sequestration downstream; these vessels can contain both parasitized and uninfected red bloods (200X, H&E). The classic appearance of a ring hemorrhage with fibrin **(D)** is shown; these hemorrhages can also include pigmented parasites, free pigment, and admixed fibrin within the microvessel at the nexus of the lesion; uninfected erythrocytes constituting the surrounding hemorrhage are seen (400X, H&E). Two examples of sequestration showing predominantly early (less pigmented) parasites **(E)**, and late stage (more pigmented) parasites **(F)** densely packing vessels (1000X, H&E).

**Figure 4:**
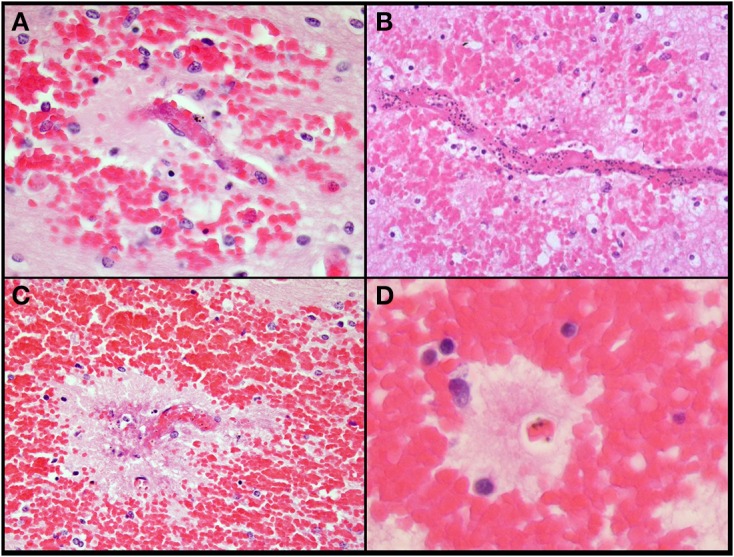
**Additional images of ring hemorrhages which highlights the presence of fibrin **(A)**, parasites **(B,C)**, pigment **(A–C)**, rarified brain **(B–D)**, and variable-sized hemorrhages**. All images are originally 600x, H&E.

*Ring hemorrhages* were present on the surface of the cerebellum and within the cerebral and cerebellar white matter in all patients with CM2 (Figures 2C–F, Figures 3A–D, and Figure 4). To a lesser degree, rare scattered ring hemorrhages could be seen in the cerebellum of CM1 patients. Discoloration and ring hemorrhages were never seen in SMA, CM3, or controls (Figure 2B).

*Sequestration* of PRBCs occurred in CNS capillaries in all CM1 and CM2 cases, but by definition rarely if at all in SMA, CM3, or control cases (Figures 3C–F). Pre-capillary arterioles and/or post-capillary venules contained PRBCs, often showing a margination pattern with a clear central lumen. The surrounding capillaries often appeared plugged or completely occluded. Meningeal capillaries rarely showed evidence of sequestration. Sequestration of PRBCs was present to some degree in all parts of the brain sampled and was most dense in cerebral cortex sites and cerebellum folia; however, there were no statistically significant differences in sequestration between these sites (Milner et al., 2013a). There was no difference between the degree of sequestration or pigment accumulation between cortical gray matter and white matter in either the cerebrum or the cerebellum (Milner et al., 2013a). CM1 cases displayed primarily early pigmented trophozoites, best visualized on smear preparations (Milner et al., 2012). CM2 cases displayed larger, more heavily pigmented later stage trophozoites and schizonts. CM3, SMA and control cases showed limited, scattered parasite elements that were predominantly free pigment globules (remnants of the rupture of schizont-containing erythrocytes). Extra-erythrocytic hemozoin pigment (free globules within vessels or within macrophages) was by definition most prominent in CM2 cases, where it was abundant in the cerebral cortex sites and cerebellar folia. Intravascular accumulation of hemozoin-laden macrophages was seen in many cases of CM2, less commonly in CM1, and rarely in CM3, SMA, or controls.

In contrast to patterns of sequestration in the cerebral hemispheres, perivascular *hemorrhages* only occurred in white matter. Hemorrhages were abundant in all layers of the cerebellum including the molecular layer, Purkinje cell layer, granule cell layer and deep cerebellar white matter (Figure 3B). All other CNS sites contained hemorrhages, but to a lesser degree than in cerebrum or cerebellum. Most of the hemorrhages contained a central distended capillary containing fibrin (Figures 3D, 4). The extravasated erythrocytes, in hemorrhages, were composed almost exclusively of non-parasitized RBCs (Figures 3D, 4). Additional examples of ring hemorrhage pathology are found in Supplemental File 2. Thrombi were often seen without associated hemorrhages. In the cerebrum, most thrombi occurred in the white matter, but some were noted in gray matter. All CM2 cases displayed capillary thrombi; these were present only rarely in CM1 and not seen in cases classified as CM3 or SMA, although detailed analysis revealed disordered coagulation in the majority of CM patients overall (Moxon et al., 2013). Only a single control case with clinical sepsis had microthrombi consistent with disseminated intravascular coagulation.

*Durck's granuloma*, an area of rarefied brain containing activated microglial/macrophages appearing at the site of a prior ring hemorrhage, was rare (2 cases) and seen only in CM2 cases with prolonged in-hospital survival (>45 h, compared to the median for CM2 cases, which was 18 h) (Milner et al., 2012).

*No inflammatory cell infiltrates or infarcts* within brain parenchyma were seen in any cases of HCM. Control cases with encephalitis or meningoencephalitis contained perivascular lymphoplasmacytic infiltrates in the space of Virchow.

Grossly identifiable *brain swelling* was present in all CCM cases and the majority of controls, the degree of swelling ranging from moderate to severe. Mean brain weight to body weight ratios were similar across all groups CM1 cases (0.09) compared to CM2 (0.10), CM3 (0.10), and controls (0.11) consistent with the gross presence of brain swelling in most cases (although no standards are available for comparison in this study). Herniation of either cerebellar tonsils into the foramen magnum or >3 mm of the uncinate process of the temporal lobes over the tentorium with a prominent notch or groove was not common and only convincingly noted in 24 cases (CM1 = 3, CM2 = 9, CM3 = 7, controls = 5). Depression of the mammillary bodies more than 5 mm below Greenhall's line, indicative of brain swelling, was common overall (53 of 75 cases in which it was assessed); this sign was present in 7 of 10 (70%) CM1 cases, 23 of 29 (79%) CM2 cases, 12 of 19 (63%) CM3 cases, 4 of 4 SMA cases (100%), 6 of 12 (50%) non-malaria comas, and 1 of 1 incidental parasitemia (non-comatose patient) (Esiri and Oppenheimer, 1989).

**Figure 5:**
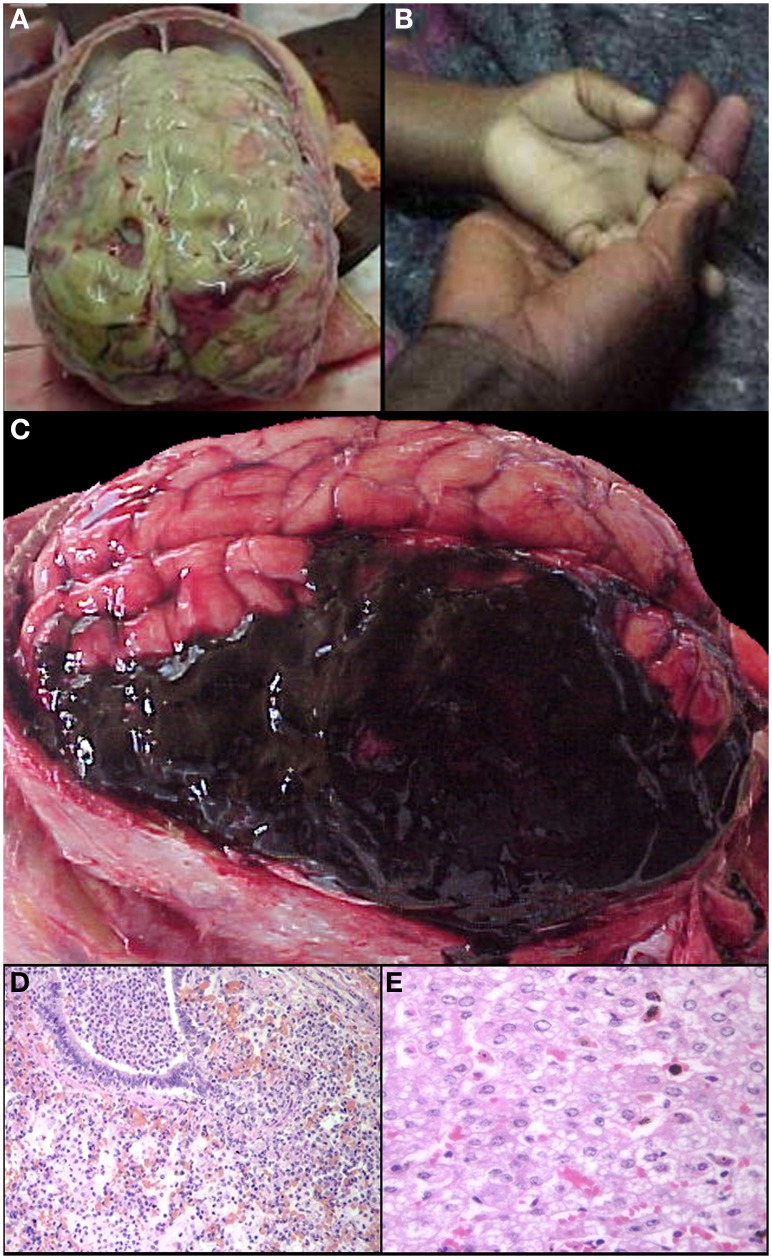
**A summary of representative images from the gross and histological examinations of the brain and other organs in the autopsy series is shown for control and non-CM cases**. Meningitis **(A)**, one of the exclusion criteria for the clinical case definition, and severe malaria anemia **(B**—clinical photograph of a child's hand in mother's hand at admission) served as controls in the study. Intracranial hemorrhages **(C)** were a cause of death in several patients who met the clinical case definition for CM but without brain sequestration. Other causes of death in patients meeting the clinical case definition of CM but without evidence of brain sequestration included pneumonia **(D)** and Reye's syndrome **(E** Whitten et al., 2011).

The non-malaria comas with infectious etiologies included meningitis or meningoencephalitis due to *Haemophilus influenza*, *Mycobacterium tuberculosis*, or *Streptococcus pneumoniae* (commonly associated with pneumonia) (Figures 5A,D). With the exception of scattered sequestration in a few organs, the primary finding in the clinical SMA patients was marked anemia (Figure 5B). In CCM patients who had no evidence of sequestration in the brain at autopsy (CM3), the CNS pathology included intracranial hemorrhage with and without congenital vascular malformations (Figure 5C), benign cortical cysts, viral encephalitis, and meningoencephalitis. Reye's syndrome was found to be a cause of coma in 5 cases of clinically defined cerebral malaria without evidence of sequestration (Figure 5E) (Whitten et al., 2011).

#### Cardiovascular pathology

**Figure 6:**
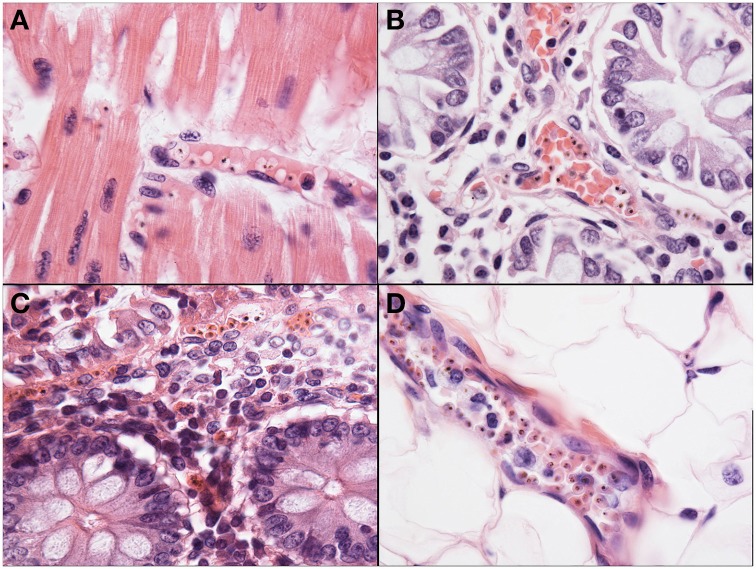
Representative images of pathology outside of the brain are shown. The heart **(A)** was a common site of sequestration and parasites could consistently be found in CM patients (1000X, H&E). The small bowel **(B)**, including jejunum and ileum, as well as the stomach and colon **(C)** were common sites for sequestration in all patients, regardless of diagnosis, although the most intense sequestration in the gastrointestinal tract was in CM patients (400X, H&E). The subcutaneous adipose layer of the skin **(D)** was a common site of dense sequestration but, unlike the gastrointestinal tract, was only seen in cases with CM.

Sequestration of PRBCs in the heart was observed in less than half of the CM cases, in both CM1 (46%) and CM2 (40%) categories. The degree of cardiac sequestration was less impressive than the sequestration in other organs from CM1 and CM2 cases (Figure 6A). There was no associated cardiac myofiber necrosis or inflammation, or other evidence of myocardial injury, and there were no differences in histopathology between the right and left ventricular myocardium. In a few cases of CM 3, SMA, and controls, sparsely scattered parasites were observed.

#### Respiratory pathology

**Figure 7:**
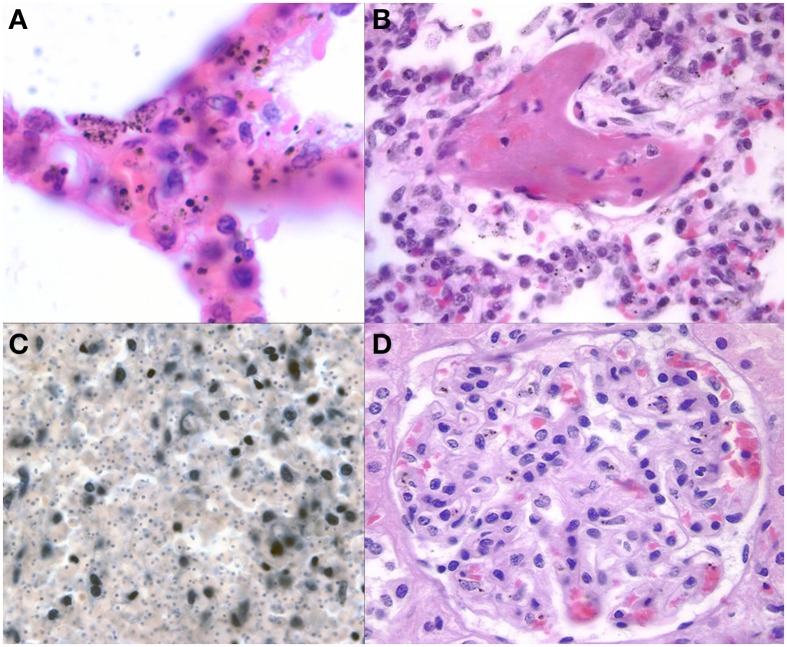
**Representative images of pathology outside of the brain are shown**. The lungs **(A)** demonstrate some sequestration of parasites but more frequently have heavily burdened macrophages with variable size pigment globules (1000X, H&E). Fibrin thrombi **(B)** were present in a subset of CM2 and other cases in the lungs suggestive of DIC (400X, H&E). The spleen **(C)** in a patient with CM after depigmentation of the section using picric acid followed by Giemsa stain (from Milner, unpublished data) demonstrating the unique finding of dense parasite burdens in CM1 spleens (200X, Giemsa). A representative glomerulus **(D)** from the kidney shows malaria pigment within macrophages and microthrombi present in capillaries (400X, H&E).

The presence of parasites in the lungs was associated with cerebral sequestration but the degree of sequestration seen in the brain was more intense than in the pulmonary vasculature. An intravascular accumulation of leukocytes, mostly of the monocyte/macrophage lineage, was observed in all cases, regardless of diagnosis (Figure 7A). These inflammatory cells occupied distended capillaries. No infiltrates were seen in the interstitial space. The presence of malaria pigment within the monocyte/macrophages was significantly more common in both CM1 and CM2 than in SMA, CM3, or controls (Milner et al., 2013b). Acute pneumonitis of presumed bacterial etiology, either bronchocentric or lobar, was observed in 27% of CM1, 21% of CM2, 25% of SMA, 63% of CM3, and 56% of controls (Milner et al., 2013b). Histological evidence of pulmonary edema was present in all groups and at approximately the same rate (40%) except in SMA where it was not seen (Milner et al., 2013b). Capillary microthrombi (Figure 7B) were seen most commonly in CM2 cases; none was observed in the CM1 and SMA cases. In CM2, CM3 and controls, the proportion of cases with capillaries containing microthrombi were 31, 6, and 22%, respectively. Other findings in the lungs included granulomata due to *Schistosoma* spp eggs (2), granulomatous tuberculous pneumonitis (1), lymphoid interstitial pneumonitis (observed in 10 patients, 6 of whom were HIV positive), and bronchiolitis obliterans with organizing pneumonia (3). Intracapillary megakaryocytes, a non-specific stress response, were present in most cases.

#### Gastrointestinal pathology

Sequestration of PRBCs occurred with great frequency and in large numbers in the microcirculation of the gut of the HCM (Figures 6B,C). The accumulation of PRBCs was almost exclusively in the most superficial mucosal capillaries, and was not associated with ulcers or obvious changes other than congestion. Parasites remained clearly visible in the gut capillaries even when the mucosal epithelium exhibited autolysis after death. Sequestration occurred in all gut locations studied, with stomach, small bowel, and colon roughly equally involved. Gut associated organs, such as sub-mandibular salivary gland and pancreas, did not show significant sequestration, but did occasionally have small numbers of PRBCs. Gut sequestration correlated with histologically defined categories; 75% of CM1 and 63% of CM2 had significant levels of sequestration. Few CM 3 cases had sequestration (5–22% depending on segment of gut) and it was noted in only one case of SMA. Control cases did not have sequestration in the gut.

Intussusceptions of small bowel were common, and were statistically significantly associated with HCM (55% of HCM cases vs. 28% of controls, *p*-value < 0.05). There was a statistically significant association between intussusceptions and parasite counts in the gut, but intussusceptions were not associated with lymphoid hyperplasia (data not shown).

No hemorrhage, necrosis, architectural abnormality (such as villous blunting of small bowel), or abnormal inflammatory cell infiltrate were noted in any gut location in association with sequestration.

#### Hepatobilliary pathology

Sequestration in liver sinusoids was not encountered in Malawian children. PRBCs could be found in sinusoids in very small numbers in most HCM cases but the morphology and location were suggestive of circulating peripheral blood forms; margination or filling of sinusoids by PRBCs was not seen. There were similar findings in the other patient groups, irrespective of the presence or absence of peripheral parasitemia. Increased numbers of Kupffer cells/macrophages were invariably present in CM1 and CM2 cases, and were also present in most CM3 cases. Kupffer cell hyperplasia was also seen in more than half of controls, although to a subjectively lesser degree than in HCM cases. The Kupffer cells were always heavily laden with hemozoin. In HCM, the Kupffer cells were distributed throughout the liver parenchyma with some collections in the portal triads. In the parasitemic non-HCM cases, the bulk of the Kupffer cells and free pigment present were within portal triads as large collections with little scattered throughout the parenchyma (Whitten et al., 2011). Hepatocyte necrosis was rare in all groups except SMA, and when present, was sparse and subtle, usually lobular spotty necrosis and no periportal distribution. Thrombi were seen in only one case, despite their abundance in the brain and findings in other organs including gut, lung, and kidney in the CM 2 cases. Previously, we reported that 10 control cases showed microvesicular fat and had a clinicopathological picture consistent with Reye's syndrome (Figure 5E) (Whitten et al., 2011). Inflammation was minimal overall with no differences in degree or location of inflammatory cell infiltrates between CM1, 2, 3 and control cases. Several patients had incidental *Schistosoma* spp eggs. Hepatocytes in the post-sporozoite stage of infection (i.e., liver schizonts with various numbers of merozoites) were not seen. The gall bladder mucosa was severely autolyzed in all cases and could not be assessed objectively for the presence of sequestered parasites.

#### Genitourinary pathology

Only five of 41 cases of CM showed dense renal vascular PRBC sequestration in our series. In these cases, sequestration of PRBCs occurred in glomerular and interstitial capillaries. Glomerular capillaries usually had only small numbers of hemozoin laden macrophages present (Figure 7D). No interstitial or glomerular inflammatory cell infiltrates were present. Pigmented casts, composed of sloughed cells/pigment accumulated in tubules and discharged into urine, were not seen. Four cases (3 CM2 and 1 CM3) showed precipitation of a calcium-like mineral within and immediately adjacent to distal convoluted tubules. No other glomerular changes were noted by light microscopy. Microthrombi were present in glomerular capillaries in about half of CM 2 cases. No sequestration of PRBCs was seen in the bladder mucosa or wall in any case.

#### Reproductive and endocrine pathology

Mild accumulation of PRBCs was seen in the gonads but in only 20% of CM1 and 11% of CM2 cases. The degree of sequestration was less than seen in other organs. Thyroid and adrenal gland were unremarkable with only very rare examples of sequestration, and always in CM1, CM2, or SMA patients. The pituitary in one case showed a striking pattern of sequestration in the neurohypophysis akin to the brain, but the adenohypophysis in the same patient was devoid of parasites.

#### Hematological pathology

Disseminated intravascular coagulation (DIC, defined pathologically as capillary microthrombi occurring in two or more organs) was observed frequently, predominantly in CM2 cases (25/40, 63%). The most commonly involved organs were CNS, lung, kidney, and bowel mucosa. DIC was seen in less than 10% of all other diagnostic categories. The spleen was enlarged in most cases, and there was variation in the ratio of spleen weight to other anthropomorphic measures but the difference was not significant between groups (data not shown). Microscopically, the spleen showed abundant malaria pigment in macrophages and some free pigment in nearly all cases including (although significantly less) most controls. Most of the pigment was in the red pulp, but the lymphoid follicles of the white pulp occasionally contained pigment. After de-pigmentation of sections in picric acid, individual PRBCs were sometimes noted on careful examination but large numbers were a feature only of CM1 patients (Figure 7C). No splenic infarcts were noted and no case of splenic rupture was identified. Bone marrow consistently harbored malaria pigment in macrophages, and to a lesser degree, apparent free pigment. The quantity of pigment was always less than that in liver or spleen. A minor degree of PRBC sequestration was noted in marrow capillaries in CM1 and CM2 cases, but was not always present. Depletion of hematopoietic cells was not noted. Megakaryocytes were always present in adequate numbers despite the consistent thrombocytopenia in all cases of CM1 and 2 (Taylor et al., 2004). Lymph nodes rarely contained PRBCs and never showed significant sequestration. The thymus showed cortical depletion of lymphocytes in many cases, roughly correlating with the length and severity of illness described clinically. No sequestration was present.

#### Skin and soft tissues pathology

Sequestration of PRBCs in the skin only occurred in the capillaries and small venules and arterioles of the subcutaneous adipose tissue, sparing the dermis. Sequestration was less dense than in the CNS with fewer PRBCs, and it was restricted largely to the cases of CM1 and CM2 (Figure 6D). No hemorrhages or areas of necrosis were present and obvious microthrombi were not noted, even in cases with disseminated intravascular coagulation. No sequestration or even vascular congestion was noted in skeletal muscle despite our previous observations of prominent inducible nitric oxide synthase and migration inhibitory factor production in skeletal muscle (Clark et al., 2003a). Sequestration of PRBCs was not noted in adipose tissue except in subcutaneous fat and in brown fat, which was more abundant in the younger cases. No sequestration and no other abnormalities were noted in brachial plexus or other peripheral nerves in any of the cases.

#### Qualitative assessment of sequestration

**Figure 8:**
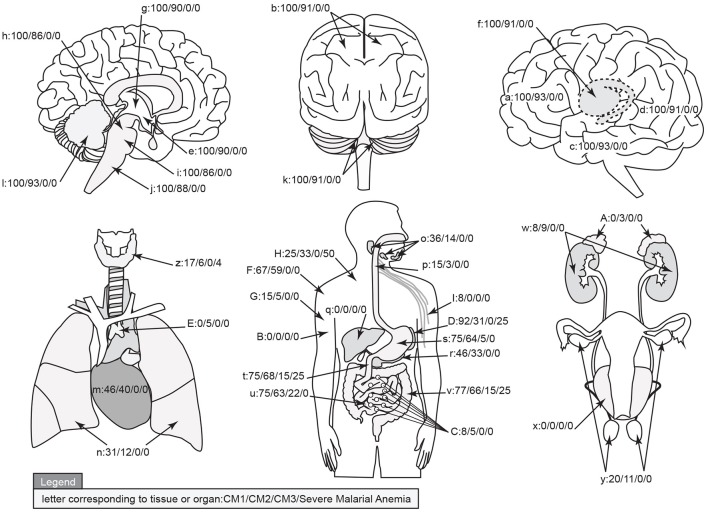
**A graphical map of the distribution of parasite sequestration in all organs studied showing the percentages of cases in which sequestration was found for each of the diagnostic categories CM1/CM2/CM3/SMA (where organs were available)**. For this figure, the definition of “sequestration” was that more than 50% of the red blood cells in any capillary, pre-capillary arteriole, or post-capillary venule were parasitized or that showed a continuous layer of cytoadherent parasitized red blood cells contiguous with the vascular endothelium present in the tissue. The organs by key are given in Table 2. Image professionally provided by Anet James/Gallery 55 (anetj@yahoo.com).

The organ sampled for each autopsy as described above are graphically represented in Figure 8 and marked with the % of cases having sequestered pigmented or unpigmented parasites present in the vessels. Note that CM1 has 100% of vessels containing parasites while CM2 ranges from 86 to 93% by site. The CM2 patients always had pigment (either as free pigment globules or within macrophages), ring hemorrhages, and obvious fibrin thrombi scattered throughout [elements which are counted in the quantitative methods described previously when determining histological cerebral malaria (Milner et al., 2013a)]. The data for all organs are summarized in Table 2. The gastrointestinal tract and the spleen were strongly associated with CM1 & CM2 and to a lesser degree in SMA. Only gastrointestinal sequestration was a feature of CM3. Non-Malaria Comas and Incidental Parasitemia cases did not show sequestration.

**Table 2:** **The qualitative totals for absent/present sequestration (%) by organ system and by individual organ across the diagnostic categories are provided**.

	**Organ/System**		**CM1**	**CM2**	**Severe Malarial Anemia**	**CM3**	**Non-malaria Coma**	**Incidental parasitemia**	**Key (Figure 8)**
Brain	Telencephalon	Frontal lobe	100	93	0	0	0	0	a
		Parietal lobe	100	91	0	0	0	0	b
		Temporal lobe	100	93	0	0	0	0	c
		Occipital lobe	100	91	0	0	0	0	d
	Diencephalon	Hippocampus	100	90	0	0	0	0	e
		Caudate lobe	100	91	0	0	0	0	f
		Thalamus	100	90	0	0	0	0	g
		Midbrain	100	86	0	0	0	0	h
	Hindbrain	Pons	100	86	0	0	0	0	i
		Medulla	100	88	0	0	0	0	j
		Cerebellar tonsils	100	91	0	0	0	0	k
		Cerebellar dentate	100	93	0	0	0	0	l
	Thorax	Heart	46	40	0	0	0	0	m
		Lung	31	12	0	0	0	0	n
	Gastrointestinal Tract	Salivary gland	36	14	0	0	0	0	o
		Esophagus	15	3	0	0	0	0	p
		Liver	0	0	0	0	0	0	q
		Pancreas	46	33	0	0	0	0	r
		Stomach	75	64	0	5	0	0	s
		Jejunum	75	68	25	15	0	0	t
		Ileum	75	63	0	22	0	0	u
		Colon	77	66	25	15	0	0	v
	Genitourinary	Kidney	8	9	0	0	0	0	w
		Bladder	0	0	0	0	0	0	x
		Gonad	20	11	0	0	0	0	y
	Endocrine	Thyroid	17	6	4	0	0	0	z
		Adrenal	0	3	0	0	0	0	A
	Reticulo-endothelial	Bone marrow	0	0	0	0	0	0	B
		Lymph node	8	5	0	0	0	0	C
		Spleen	92	31	25	0	0	0	D
		Thymus	0	5	0	0	0	0	E
	Soft Tissue	Skin	67	59	0	0	0	0	F
		Skeletal Muscle	15	5	0	0	0	0	G
		Brown adipose	25	33	50	0	0	0	H
		Peripheral nerve	8	0	0	0	0	0	I

The key to the pictographic representation in Figure 8 is given in the last column.

## Discussion

During the twentieth century, fewer than 350 cases were included in the published descriptions of the histopathological manifestations of severe malaria, and of these, fewer than 45 cases were children. Histopathological studies laid the groundwork for our understanding of the pathogenesis of cerebral malaria, but the interpretation of all were limited by the lack of prospective collection and correlation with clinical data. More importantly, the previously published work did not address the pathology in the group bearing the brunt of the malaria burden, the children of sub-Saharan Africa. Our study provides modern, thorough, clinical and pathological data on prospectively characterized pediatric cerebral malaria cases.

Sophie Spitz described the morphological findings in the CNS of non-immune adults dying of malaria (Spitz, 1946). She found more Durck's granulomata than we did, but otherwise her description of the basic light microscopical changes were nearly identical to our findings. We have noted previously, and continue to see in this larger cohort, the distinction between CM1 and CM2 on histopathology; patients die at different time points in the life-cycles of their asexual parasites, suggesting that no single stage affords an essential immediate trigger of death. CM1, the less common pattern, appears to represent an overall more rapidly progressive disease with higher levels of parasite burden in the spleen (the primary organ of parasite clearance) but little end organ pathology (i.e., ring hemorrhages). In contrast, the pathology associated with the CM2, the more common of the two, appears to be associated with a more prolonged illness with the development of end organ pathology in the brain. In some cases, this pathology may even begin to resolve (indicated by the presence of Durck's granuloma in a few cases and the variability of brain sequestration in CM2 cases despite the presence of ring hemorrhages) before the child dies. The CM2 cases tend to have more pigment in various organs than is found in CM1, suggesting that in CM2, the patient had experienced multiple recent rounds of the parasite life cycle.

A dilated capillary containing a microthrombus was at the center of nearly all ring hemorrhages, being especially likely to be identified if serial sections were performed. In most hemorrhages, the ring of blood extends from the vessel; however, in many there is a zone immediately around the vessel in which there are no red blood cells. This pattern suggests that, in vessels containing cytoadherent parasitized red cells, breakdown of the endothelial barrier occurs, uninfected red blood cells flow into tissue, a thrombus forms closing the damaged vessel, and cells stop flowing into the parenchyma. It is not possible to determine whether the formation of a smaller microthrombus contributes to the initial vessel wall damage although abundant thrombi without hemorrhages were present (Moxon et al., 2013). We did not see enough Durck's granulomata to be able to deduce a sequence of events between blood-brain-barrier breakdown and the formation of these lesions, which are believed to result from the resolution of microhemorrhages (Moxon et al., 2013).

Although brain swelling was usual in all cases, we detected herniation of the brain in only a minority of cases, and necrosis of herniated tissue in none. Herniation was most frequently found in CM2 cases (~25%). Most patients with CM (CM1 and 2) die of a respiratory arrest. Injury of the respiratory center may result from rapid brain swelling even if there is insufficient time for frank, fixed herniation to develop. We did not see signs in the respiratory nuclei in the pons typically associated with more long standing herniation (necrosis and hemorrhages). Our current clinicoradiological data indicate that brain swelling is common with CM, and that it is strongly associated with a fatal outcome (Potchen et al., 2010, 2012).

Spitz (1946) described more extensive cardiac sequestration in adult soldiers than we saw in Malawian children. She identified myocardial edema in 84%, but this was not a prominent feature in our cases. She also reported interstitial myocardial mononuclear inflammation in 20/50 cases and pericarditis in one. We saw no inflammatory cell infiltrates in any case. Antimalarial drugs can be cardiotoxic (White, 2007) but we saw no evidence of myofiber injury. High levels of circulating tumor necrosis factor-α (TNF) can induce myofiber apoptosis in mice (Kubota et al., 2001), but we saw no apoptosis in myofibers or endothelial cell*s* in the children in this series.

Spitz (1946) found that, in non-immune adults, pulmonary vascular sequestration paralleled that seen in the brain (i.e., was abundant and common) in contrast to our data showing significant lung sequestration in only 9 of 55 cases of CM. She also reported pulmonary edema in all of her cases, whereas we found it in 20 of 55 CM1-2 (36%). Pneumonia (defined as neutrophils in contiguous alveolar spaces) is a frequent complication in our pediatric population, similar to the pneumonia commonly seen in adults (Applebaum, 1944; Spitz, 1946; Taylor et al., 2006). Spitz found pneumonia in 42%, and we found it in 22% of CM1 and 21% of CM2 cases. It was more frequent (63%) in CM3, and was often severe enough to be considered to be the cause of death for those patients. All of the CM3 cases had abundant hemozoin pigment in reticuloendothelial organs, indicating recent and possibly frequent malaria infections. The malaria infection in CM3 patients may have contributed or predisposed to the illness that caused the patient's death, or may have been incidental and a reflection of the background prevalence of *P. falciparum* infections in this community. The levels of sequestration found in the lungs of CM3 patients are considerably less than those found in the lungs of patients with cerebral sequestration. There was no interstitial inflammatory cell infiltrate resembling that typically seen in fatal pulmonary viral infection. In contrast to adults, we did not see features of Acute Respiratory Distress Syndrome (i.e., diffuse alveolar damage—hyaline membranes, enlarged reactive pneumocytes, intravascular neutrophil accumulations) in our cases (Tomashefski, 2000). Most of the deaths were respiratory arrests, but these appear to be due to a centrally mediated cessation of breathing rather than respiratory failure due to an anatomic pulmonary lesion (Potchen et al., 2010, 2012, 2013; Kampondeni et al., 2013).

Spitz reported gut sequestration in 15 of 22 cases and considered it parallel to the degree of sequestration in other organs (Spitz, 1946). Olsson described sequestration in only one of 20 adult soldiers with acute malaria in Vietnam on small bowel biopsy (Olsson and Johnston, 1969). The prevalence of significant gut sequestration might explain the abundant diarrhea and malabsorption seen particularly in young children with malaria infections although diarrhea was not common in our case series (Karney and Tong, 1972). Given the large surface area in the bowel and the voluminous capillary beds, it is likely that a greater part of total-body sequestration of parasites occurs here (Seydel et al., 2006). The gut may be an important site of sequestration in non-fatal and asymptomatic as well as severe malaria and the large number of inflammatory cells in the lamina propria may contribute to the levels of systemic cytokine activation observed in malaria infections with different levels of severity.

In the kidneys, Spitz (1946) described PRBCs and haemozoin-laden macrophages in peritubular capillaries but not PRBCs in glomerular vessels. Pigment was seen in 60% of cases in the distal convoluted tubules, but only 14% had clinical “blackwater fever.” Glomerular hypercellularity was seen in 18% of those cases. In contrast, we find few PRBCs in any vessels, occasional haemozoin-laden macrophages in glomeruli and no casts. “Blackwater fever” was not seen in any of these patients during life. We did encounter a high number of capillary microthrombi in CM2 patients, consistent with the hypothesis that a subset of these patients is experiencing DIC. Acute renal failure occurs in both adults and children, partially immune and non-immune, but is more prevalent and severe in non-immune/semi-immune patients. Malawian children in our cohort have various degrees of partial but incomplete immunity (unlike older adolescents and adults); oliguria and/or kidney injury were not encountered in any of the patients in our series.

Disseminated intravascular coagulation, a systemic coagulopathy, is common in CM2 cases; organs where we found the thrombi indicative of DIC included cerebral, pulmonary and renal capillaries. There is little morphological alteration in the bone marrow, lymph nodes or thymus. Splenic congestion and pigment accumulation were prominent, but the spleens in malaria cases were not significantly larger than in controls (all patients come from a background population of endemic malaria and some degree of splenomegaly is very common). Massive splenomegaly was not present in our series, although children and adults with hyper-reactive malarial splenomegaly are encountered in clinical practice in Malawi (Bedu-Addo and Bates, 2002). In contrast to the Spitz study, the spleen does not appear to represent a location of significant sequestration of viable parasites within RBCs, but rather, for the vast majority of our cases, it appears to be the site for clearing effete RBCs containing dead or dying parasites. No schizonts were noted there.

Our group has previously shown evidence of parasite pLDH in the skin (Seydel et al., 2006), and the autopsy findings confirm that there is significant subcutaneous adipose tissue sequestration in the majority of cases of CM. The absence of skin sequestration in our control cases and in those with CM3 resulted in a high negative predictive value for these specimens. This finding corroborates others (Nakazawa et al., 1995; Wilairatana et al., 2000), suggesting that measuring subcutaneous sequestration of PRBCs by fine needle aspiration may aid in diagnosing and following severe malaria, especially when peripheral parasitemia declines with treatment. We saw no necrosis or gangrene of skin (Gopinathan and Bhalla, 1987; Sharma, 1987; Kumar et al., 1995). Skin sequestration may make little contribution to cytokine production as the cutaneous mononuclear cells are predominantly dendritic cells and are largely confined to the epidermis, where sequestration does not occur.

The sequestration of *P. falciparum*-infected erythrocytes occurs in many different organ systems in the human host. When considered together, specific organ patterns of disease suggest that several different pathogenic mechanisms could converge to produce the clinical syndrome of cerebral malaria. Increased brain volume appears to be nearly universal, but it may not always be sufficient for the cause of death with either degree of increase or rate of development being important considerations. Other potential contributors in our cohort include the systemic activation of coagulation and co-infections like pneumonia (Milner et al., 2013b; Moxon et al., 2013). The etiologies of increased brain volume may include true cerebral edema (either vasogenic or cytotoxic), vascular congestion, and increased cerebral blood flow volume. Anti-malarial medications working in concert with the immune response are currently the only approach to reducing the parasite burden; ancillary measures aimed at reducing brain volume or mitigating its effect should be investigated as a priority. Additional studies to evaluate coagulation status and co-infections may also be important for tailoring treatment and management.

We have shown that patients with HCM have a high burden of sequestration in many organs of the body, including the brain. Parasitemic patients who do not meet our modified definition of CCM (i.e., who do not have malaria retinopathy) have little to no burden of sequestered parasites.

## Funding support

This work was supported by the U.S. National Institutes of Health (RO1 AO34969, K23 AI072033) and by The Wellcome Trust, UK (042390/Z/94).

### Conflict of interest statement

The authors declare that the research was conducted in the absence of any commercial or financial relationships that could be construed as a potential conflict of interest.
